# Herpes simplex virus 1 as an oncolytic viral therapy for refractory cancers

**DOI:** 10.3389/fonc.2022.940019

**Published:** 2022-07-27

**Authors:** Hayle Scanlan, Zachary Coffman, Jeffrey Bettencourt, Timothy Shipley, Debra E. Bramblett

**Affiliations:** ^1^Rowan School of Medicine, RowanSOM-Jefferson Health-Virtua Our Lady of Lourdes Hospital, Stratford, NJ, United States; ^2^Monroe Clinic Rural Family Medicine Program, The University of Illinois College of Medicine Rockford, Monroe, WI, United States; ^3^Department of Biomedical Sciences, Burrell College of Osteopathic Medicine, Las Cruces, NM, United States; ^4^Department of Biomedical Sciences, A.T. Still University School of Osteopathic Medicine in Arizona, Mesa, AZ, United States

**Keywords:** oncolytic virus, Herpes simplex virus 1, cancer, T-VEC, virotherapy

## Abstract

The need for efficacious and non-toxic cancer therapies is paramount. Oncolytic viruses (OVs) are showing great promise and are introducing new possibilities in cancer treatment with their ability to selectively infect tumor cells and trigger antitumor immune responses. Herpes Simplex Virus 1 (HSV-1) is a commonly selected OV candidate due to its large genome, relative safety profile, and ability to infect a variety of cell types. Talimogene laherparevec (T-VEC) is an HSV-1-derived OV variant and the first and only OV therapy currently approved for clinical use by the United States Food and Drug Administration (FDA). This review provides a concise description of HSV-1 as an OV candidate and the genomic organization of T-VEC. Furthermore, this review focuses on the advantages and limitations in the use of T-VEC compared to other HSV-1 OV variants currently in clinical trials. In addition, approaches for future directions of HSV-1 OVs as cancer therapy is discussed.

## Introduction

Nearly 40% of people in the United States will be diagnosed with cancer during their lifetime (1). In 2018, the CDC attributed 21.1% of total deaths to malignant neoplasms which can become unresponsive to treatment (refractory) (2). Current anticancer drugs are toxic and often not entirely effective. Therefore, there is an urgent need for novel therapies that are efficacious and non-toxic. New treatments involving the use of oncolytic virus (OV) therapies, many of which are currently undergoing clinical trials, are showing great promise.

### Herpesviridae properties

**Figure 1:**
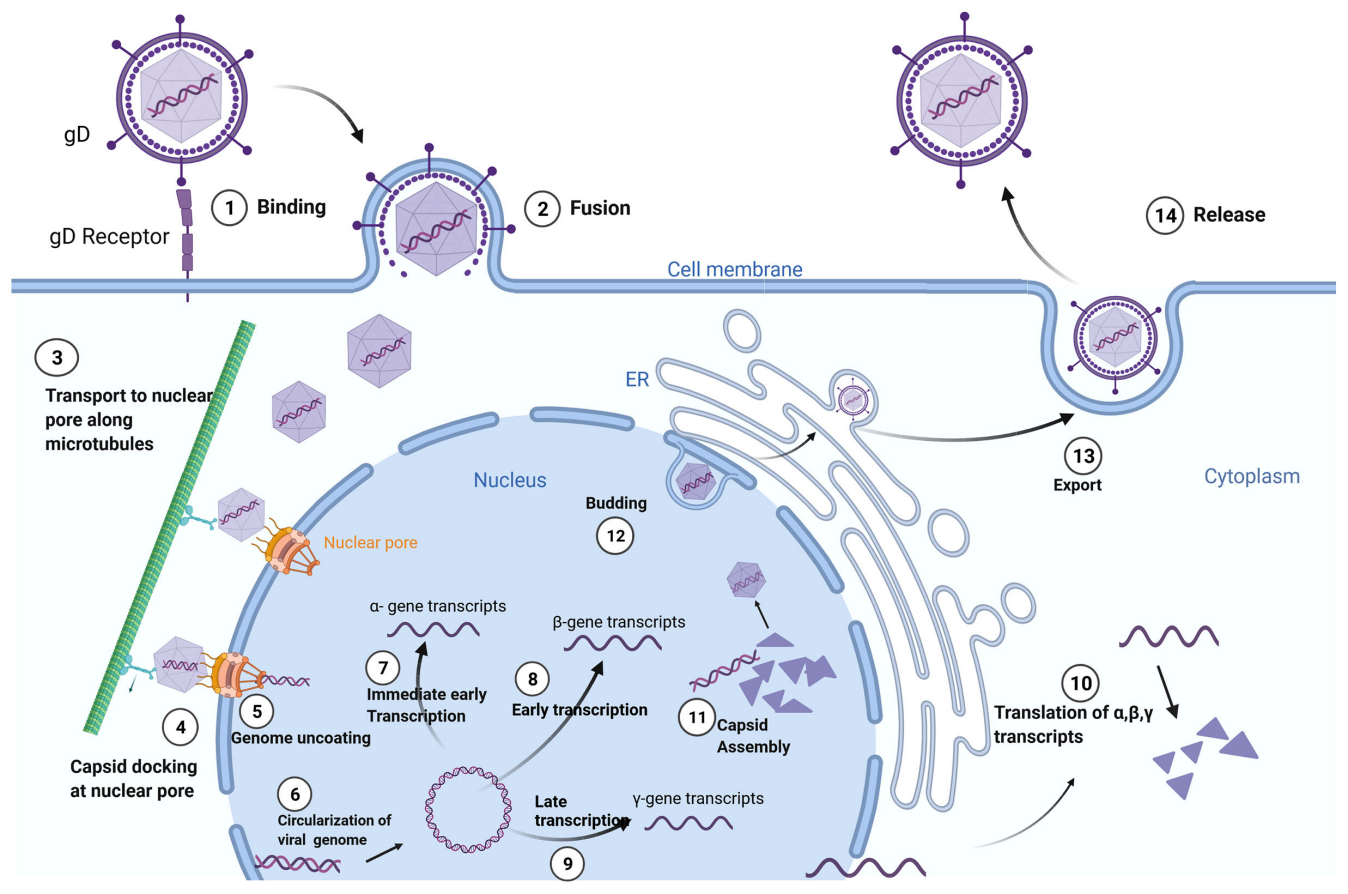
Herpes Simplex Virus-1 (HSV-1) Life Cycle: Adsorption and fusion of HSV-1 to its target cell is initiated by viral glycoprotein D (gD) to cell specific gD receptors (1). Fusion of the viral envelope with cell membrane allows for capsid entry into the cytoplasm and release of tegument proteins (2). The naked viral capsid is transported (3) to nuclear pore complexes in the nuclear envelope (4), through which the viral genome is extruded into the nucleus (5). The linear viral genome is circularized (6). Herpes viruses have three rounds of transcription: immediate early (α-genes) (7), early (β-genes) (8), and late (γ-genes) (9). Translation of the structural proteins from γ- transcripts occurs only after the initiation of viral genome replication, which is dependent on β-proteins. Viral transcripts leave the nucleus to be translated (10) in either the cytoplasm or in the context of the endoplasmic reticulum (ER). Capsids are assembled in the nucleus, encasing the viral genome in an icosahedral protein coat (11). The newly generated viral capsid acquires an envelope by budding into the inner nuclear membrane (12). The completed virus translocates through the ER and matures in the Golgi apparatus prior to exiting the cell by exocytosis (13). Created with BioRender.com.

*Herpesviridae* is a large family of enveloped, double-stranded DNA viruses that can undergo both lytic and latent lifecycles, depending on cell type infected by the virus. Cell-type specificity of the *Herpesviridae* is defined by surface glycoproteins on individual virions that interact with cell-surface receptors. Upon binding, fusion of the viral envelope with the cell membrane leads to the release of the viral capsid into the cytoplasm. The viral DNA is then transported to the cell nucleus within the now-naked nucleocapsid. The nucleocapsid then attaches to the host cell’s nuclear membrane enabling insertion of the viral genome into the nucleus through a nuclear pore. After circularization, the virus genome can be transcribed, leading to productive virus replication in permissive cells or latency in non-permissive cell types. The virus’ life cycle is complete upon budding of new virions which go on to infect neighboring cells (**Figure 1**).

**Figure 2:**
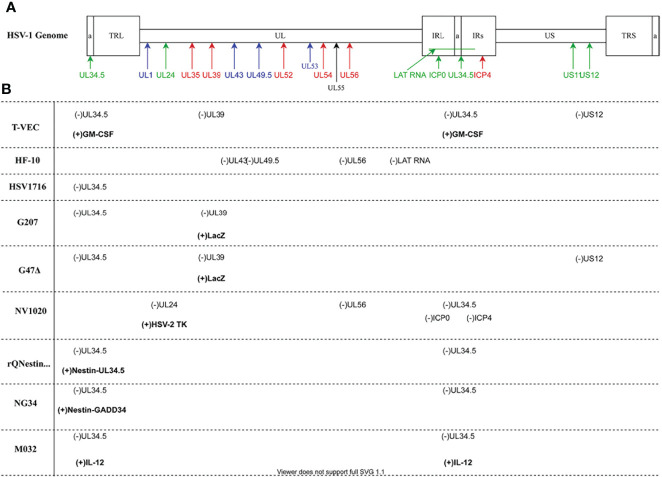
HSV-1 Genomic map and oncolytic virus modifications **(A)** The HSV-1 genome has two covalently joined segments long (L) and short (S) each of which has a unique sequence (U_L_ and U_S_) flanked by a pair of repeat sequences, the terminal and internal long repeats (TL_L_, IR_L_, TR_S_ and IR_S_). There is also a 400 base pair terminal repeat at each end of the genome and internally at the joint between the L and S segments which is called **(*a*)** Genes are coded according to functional group: blue- structural; red- replication; green- virulence. **(B)** Oncolytic viruses mentioned in the paper. Deleted viral genes are indicated as **(-)** while transgenes or viral genes that have been inserted are in bold and indicated by (+). Diagram prepared with DRAWIO.

Production of herpesvirus messenger RNA by an infected cell leads to expression of a variety of proteins that perform a wide array of functions, ranging from virion assembly to suppression of host cell antiviral responses. Gene expression occurs in three phases of transcription, and these genes have been designated by the kinetic classes, α, β, and γ, which correspond to immediate early, early, and late phases of gene expression, respectively. Proteins expressed during the α phase impact cellular and viral gene expression, including β gene expression. During the β phase of transcription, proteins involved in DNA replication are expressed, including viral DNA polymerase. Most of the γ genes encode structural proteins. Of significance to this review, gene names correspond to the region of the genome in which it resides, with the HSV-1 genome being organized into a unique long region (UL) and a unique short region (US) (**Table 1** and **Figure 2**). The designation “infected cell protein” (ICP) is given for viral proteins that are not structural.

**Table 1:** HSV-1 genes modified in OV candidates.

Protein/Gene name	Function
**ICP0/*RL2***	-Blocks IFN response; required for reactivation from latency
***UL24***	-Inhibits viral DNA sensing by the innate immune system
**ICP34.5/*UL34.5***	-Contributes to both neurovirulence and inhibition of immune-mediated clearance of the virus
**LAT RNA**	-The only transcript detectable at high levels during HSV-1 latency; promotes latency reactivation
***US11***	-Nucleolar; RNA binding protein; inhibits interferon mediated antiviral response; inhibits apoptosis; impairs autophagy
**ICP47/*US12***	-Blocks TAP loading of peptides into MHCI promoting HSV-1 proliferation
**ICP6/*UL39***	-The large subunit of the ribonucleotide reductase (R1); suppresses both Casp8-mediated apoptosis and RIPK3-mediated necroptosis
**ICP35/*UL35***	-The viral thymidine kinase
**ICP27/*UL54***	-Intermediate early protein that promotes viral DNA replication; promotes viral RNA transcription, processing, nuclear export and translation
***UL56***	-Transmembrane protein involved in vesicular trafficking; possibly contributes to transport and release of viral particles neurovirulence
**ICP4/*RS1***	-Regulates viral gene transcriptional *via* interaction with TFIID
**ICP22/*US1***	-Downregulates cellular gene expression; upregulates late viral gene expression; co-chaperone activity to promote lytic infection
**gL/*UL1***	-Envelope glycoprotein that complexes with gH*;* contributes to fusion
**gH/UL22**	-Envelope glycoprotein that complexes with gL; triggers the fusion protein gB to undergo rearrangements leading to membrane fusion
**gB/*UL27***	-Envelope glycoprotein B composes an HSV-1 spike acts as a fusion protein
***UL43***	-Envelope glycoprotein; not essential for viral entry
**gN/*UL49.5***	-Envelope glycoprotein that is not essential *In Vitro*
**gK/*UL53***	-Envelope glycoprotein; required for cell entry by binding to signal peptide peptidase (SPP)
***UL55***	-Gene dispensable for viral replication or establishment of latency; may be involved for virion assembly
**gG/*US4***	-Envelope glycoprotein that is a major antibody target
**gD/*US6***	-Envelope glycoprotein that binds to cell surface proteins HVEM, nectin-1, and nectin-2; interacts with gH/gL complex leading to fusion mediated by gB.

Green-virulence factors; Red- viral replication; Blue-viral structure.

### OV therapy

Researchers have taken advantage of the cell-killing attributes of viruses to target tumor cells specifically. One of the main advantages of OV therapy over conventional chemotherapy is that viruses are capable of self-propagation allowing them to replicate and infect more tumor cells, ideally until all cells of a malignant mass have been killed. Many virus types such as Adenovirus, Poxvirus, Coxsackievirus and Herpesvirus are being used in OV therapy development (3–12). Tumor destruction by OV therapy is accomplished by direct viral lysis of malignant cells or by indirect mechanisms involving elicitation of enhanced anti-tumor responses as the host immune system attacks the virus-infected cells, or by a combination of these two mechanisms. Advances in recombinant DNA technology and better understanding of viral pathogenesis have allowed manipulation of viral genomes, leading to improved OV specificity for tumor cell targets and greater ability to direct host responses.

The potential impact of OV therapy on patients stands in stark contrast to conventional tumor treatments. Treatment by radiation and chemotherapy typically exhibit minimal cell specificity, resulting in destruction of normal tissues in addition to the tumor targets, thus causing multiple adverse effects in patients. OV therapies offer significant advantages due to decreased toxicity stemming from their ability to specifically target malignant cells. Furthermore, OV treatments are able to circumvent resistance to conventional therapies frequently acquired by tumor cells (13).

Currently, in all stages of testing, from pre-clinical testing to phase III clinical trials, there are several Herpes Simplex Virus (HSV) variants that are being investigated for use as OV therapies (14). HSV is the most commonly investigated virus for OV development because 1) its genome is relatively easy to manipulate, 2) its surface glycoproteins can be altered to target specific cellular receptors, and 3) its replication can be controlled with herpesvirus-specific drugs such as acyclovir. One oncolytic herpesvirus therapy, Talimogene laherparevec (T-VEC; also known as OncoVEX^GM-CSF and IMLYGIC™), has received approval from the United States Food and Drug Administration (FDA) for treatment of inoperable melanoma, and investigations for its use in a wide variety of malignant conditions are underway (15).

Development of HSV-1 OVs has led to progressively more sophisticated constructs that are more refined in their oncolytic abilities. The primary focus in early studies with OVs was safety. As one of the earliest OVs brought to the clinic, G207 had few modifications to wild-type HSV-1. Importantly, its safety has been demonstrated in multiple clinical trials for treatment of many different cancer types. Later, the OV therapeutic agent, NV1020, which was first studied as a potential vaccine against HSV-2, was repurposed as an OV that could more efficiently replicate in tumor cells due to further modifications to the HSV-1 genome while maintaining minimal toxicity. In subsequent years, HSV1716, perhaps the least sophisticated of the HSV-1 OVs with respect to genomic alterations, has been used in several clinical trials with the primary outcome being demonstration of a high safety profile that ensuing OVs have striven to duplicate. With insertion of a transgene in addition to genomic modifications already in use by earlier OVs, T-VEC represents the next level of sophistication for OV-based cancer treatments. Both its efficacy and safety were such that T-VEC became the first OV treatment approved for clinical use against advanced melanoma. Currently, a plethora of trials examining the use of T-VEC in combination with other drugs and against other cancers are underway. HF10, with the greatest number of genomic alterations of the HSV-1 OVs discussed herein, was developed simultaneously with HSV1716 and T-VEC. Although genomic alterations in HF10 occurred naturally, they serendipitously imbued the OV with considerable safety and efficacy against many cancer types. The HSV-1 OV, M032, is a more recent OV variant that mirrors the development of T-VEC with the incorporation of a transgene to boost efficacy. It also shares other genomic alterations with T-VEC to minimize toxicity. Its first clinical trials are just underway. Most recently, the HSV-OV G47Δ, a derivative of G207, has been determined to be safe and effective in clinical trials in Japan. Finally, a new pinnacle of genomic engineering has been demonstrated with the OVs, rQNestin and NG34, possibly representing the future evolution of OV therapies that are specifically targeted to tumor cell type. All of these OVs will be discussed in detail in the following sections.

### HSV-1 and its potential for use in oncolytic viral therapy

The first step of cell infection by HSV-1 is adsorption and fusion between the virion and the plasma membrane of target cells, which are mucoepithelial cells and sensory neurons. Viral fusion with these cells is specifically mediated by viral glycoprotein D (gD), initially by low-affinity adherence to cell specific heparin sulfate proteoglycans (HSPGs). This is followed by high affinity binding to either nectin-1 or herpesvirus entry mediator (HVEM), the former of which is found on both epithelial and neural cells, and the latter of which is found only on epithelial cells (16).

During productive infections with HSV-1, copies of the viral genome are encased by protein nucleocapsids that leave the cell by budding. In lytic infections, cytolysis is caused by the combination of inhibition of host cell macromolecules, disruption of the host cell cytoskeleton, and induction of nuclear DNA fragmentation. The virus also induces increased membrane permeability, ultimately leading to cell death.

During acute, active infections, intracellular virus replication is followed by budding from the host cell surface, allowing the virus to infect neighboring cells. Infection of cells in oral or genital mucosae by HSV-1 often results in characteristic painful vesicular lesions, a relatively benign condition. In addition, acute infection with HSV-1 typically leads to seeding of ganglia innervating the area of the primary infection and, eventually, latency within the regional ganglia. During latency, the genome remains quiescent in the host cell, so production of new virions does not take place. Latency is established and maintained by the latency-associated transcript (LAT) (17). MicroRNAs, expressed from LAT, also block the expression of host cell genes whose expression would otherwise induce antiviral responses (18, 19). Reactivation from latency may be induced in response to a variety of triggering events, such as hormonal fluctuation, trauma, UV light, and immunosuppression, thus leading to damage of healthy tissues upon reactivation. Although mucoepithelial infections are normally benign, HSV-1 can cause herpes simplex encephalitis, an acute illness characterized by general and focal signs of cerebral dysfunction. This can lead to permanent brain damage and even death, thus presenting a significant concern for use of HSV-1 as a therapeutic agent.

In addition to the risk of neuroinvasion, there are other shortcomings associated with use of HSV-1 for OV therapy. Although intravenous delivery of cancer therapeutics is the desired delivery mode so that all metastases can be reached, treatment with HSV-1 OV is currently restricted to application by intratumoral injection. Intravenous delivery of an HSV-1 OV, however, may result in reduced amounts of virus reaching tumor sites due to virus sequestration by the large numbers of nontumor epithelial cells and/or sensory neurons displaying the herpes virus receptors, nectin-1 and herpesvirus entry mediator (HVEM). Hence, intravenous delivery of an HSV-1 OV would lead to too much of the virus non-specifically targeting healthy tissues instead of tumor tissues. Moreover, the World Health Organization (WHO) estimates that two-thirds of the world’s population under the age of 50 have been infected with HSV-1 (20). Therefore, systemic delivery of HSV-1 might also be limited by the prevalence of HSV-1-specific antibodies in the general population, which would rapidly neutralize HSV-1 OV administered intravenously.

Despite its pathogenicity and other limitations, HSV-1 has many properties that make it an attractive candidate for use in OV therapy. First, it is a DNA virus containing a large genome that does not integrate into the host genome. The virus’ genome contains several redundant elements and genes not required for infection, which along with its size, conveys the ability to engineer the viral genome for incorporation of large or multiple therapeutic gene cassettes using standard molecular cloning techniques to increase tumor specificity and improve safety. Another aspect contributing to its viability as an OV therapy is that the pathology of wild-type HSV-1 is typically mild compared to other viruses under development. Moreover, HSV-1 replication can be inhibited by members of the antiviral acyclovir drug class based on the virus’ dependence on thymidine kinase (TK), thus providing an extra layer of protection.

Other advantages of HSV-1 include its use of an envelope, which facilitates retargeting of the virus *via* genetic engineering. This is in contrast to non-enveloped icosahedral viruses such as the *Adenoviridae*, which have stringent structural constraints with respect to particle assembly. Furthermore, early and late gene expression by HSV-1 allows for sequential gene expression, providing the possibility to enhance OV efficacy. Additionally, HSV-1 utilizes multiple genes to manipulate signaling pathways to circumvent common host defense mechanisms. For example, HSV-1 inhibits innate anti-viral responses such as the interferon (IFN) response, which would otherwise result in a dramatic reduction of progeny virions produced during infection, thus limiting the effectiveness of using it as an OV. The IFN response, mediated by type I IFN and produced in response to virus infection of human cells, induces a network of host cell IFN-stimulated genes (ISGs), such as RNA-dependent protein kinase R (PKR), 2’-5’ oligoadenylate synthetase (OAS) and others that mediate cessation of replication of the virus in neighboring cells (21). Alternatively, if mounting a robust immune response against infected cells is desired in lieu of allowing HSV-1’s lytic lifecycle to run its course, manipulation of genes controlling these pathways can be readily performed in order to develop OV agents less able to evade host defenses. A summary of genes related to immune evasion abilities exhibited by wild-type HSV-1 and how they have been modulated in various strains of HSV-1 OVs is found in **Table 2**. Additionally, transgenes inserted to enhance immune responses against HSV-1 OV-infected cells are included.

**Table 2:** Genes responsible for immunomodulatory functions found in OV variants.

Affected Immune Function	Pertinent HSV-1 OV Gene	Immunomodulatory Effect of Protein Product	HSV-1 OV Variants with Modification
Antigen Presentation	UL34.5	-Inhibition of immune cell activation *via* interference with macroautophagy by infected cell -Inhibition of immune cell activation *via* interference with MHC II expression by infected cell	T-VEC, HSV1716, G207, M032
	UL47	-Inhibition of CTL activation *via* interference with TAP function	T-VEC, G47Δ
	UL49.5	-Inhibition of CTL activation *via* interference with TAP function	HF10
Inflammation	US11	-Inhibition of innate antiviral responses (enhanced expression due to deletion of UL47)	T-VEC, G47Δ
	UL24	-Inhibition of innate immune inflammatory response *via* interference with activation of NF-κB	NV1020
Inflammation	GM-CSF	-Recruitment of immune cells for enhancement of bystander effect	T-VEC
	IL-12	-Enhancement of NK cell cytolytic activity; polarization of immune response towards Th1-type response	M032

In the following sections, we review the clinically available T-VEC and compare and contrast it to other HSV OV therapies currently in clinical testing.

## Oncolytic viruses

### T-VEC

In 2015, the FDA approved T-VEC for treatment of patients with unresectable metastatic melanoma *via* intratumoral injection (12). T-VEC is a JS1 strain of HSV-1, which was selected for its ability to specifically replicate in and kill human tumor cells (22, 23). In addition to functional deletion of the wild-type HSV-1 genes, *UL34.5* and *US12* (**Figure 2**), T-VEC contains a human transgene coding for the cytokine, granulocyte macrophage-colony stimulating factor (GM-CSF) (12, 22). Preclinical studies conducted with T-VEC have demonstrated improved tumor shrinkage and clearance, and clinical studies have shown an enhanced durable response rate for advanced melanoma treatment when compared to use of GM-CSF alone (12, 22).

HSV-1 ICP34.5 is a multifactorial virulence factor encoded by *UL34.5*, which enhances virulence of the wild-type virus. In an infection by wild-type HSV-1, ICP34.5 is able to complex with protein phosphatase 1 alpha (PP1α) to dephosphorylate the alpha subunit of eukaryotic initiation factor 2 (eIF2), thus blocking the action of double-stranded RNA-activated protein kinase R (PKR) (24), which inhibits protein production. Deletion of *UL34.5*, therefore, suppresses T-VEC replication in non-tumor cells while simultaneously maintaining replication capacity in tumor cells due to the absence of PKR activity in most tumor cells (25). Thus, the deletion of *UL34.5* conveys tumor-specificity. In addition, ICP34.5 is required for the neurovirulence associated with HSV-1 (26, 27). Neurons and other cells deploy autophagy in defense against invading microorganisms (28). In this process, the host cell protein, beclin 1, stimulates autophagy in response to the PKR signaling pathway triggered by HSV-1 infection (29, 30). However, ICP34.5 binds to beclin 1, inhibiting this protective autophagy response in neurons (31). Therefore, reduced neurovirulence of T-VEC is brought about by the loss of ICP34.5 (31). ICP34.5 has also been reported to interfere with activation of adaptive immune responses in numerous ways, including inhibition of dendritic cell (DC) maturation and antigen presentation (32, 33). Specifically, ICP34.5 has been observed to block cell surface expression of MHC II antigen presentation proteins (34). In sum, ICP34.5 contributes to both neurovirulence and inhibition of immune-mediated killing of infected cells.

The efficacy of OVs can be enhanced by arming the virus so that neighboring uninfected tumor cells are killed (bystander effect) (35). This has been successfully accomplished in several virotherapies, including T-VEC, by expression of an immunostimulatory transgene. As with all OVs, productive T-VEC replication in tumor cells results in lysis of the infected cells, leading to necrotic cell death. Subsequent engulfment of the tumor antigen-containing cellular debris by host antigen presenting cells (APC), such as DCs and macrophages, leads to enhanced anti-tumor responses because the APCs can subsequently induce an adaptive immune response. This is further amplified by T-VEC because it carries the transgene, *GM-CSF*, which, when expressed, recruits an array of immune cells to the infection site and promotes maturation of DCs, macrophages, and granulocytes.

Another mechanism used by HSV-1 to block responses to viral infection involves ICP47, which inhibits adaptive immune responses by interfering with viral antigen processing by the host cell. Specifically, ICP47 is a high affinity competitor for the peptide-binding site on a host cell’s transporter-associated protein (TAP) (36, 37). TAP normally directs pathogen peptides into the host cell endoplasmic reticulum for loading onto MHC I molecules that will be transported to the cell surface for presentation to and activation of cytolytic T lymphocytes (CTL). Fully functional ICP47, therefore, restricts the immune response against the virus because viral antigen presentation by infected cells is reduced (38). Hence, deletion of the ICP47 gene (*US12*) in T-VEC enables robust CTL responses to be mounted against virus-infected cells.

A secondary consequence of deleting *US12* is the shifting of the *US11* coding region closer to the promoter that normally regulates the expression of *US12*. This shift alters its regulation so that the *US11* protein product is expressed as an early rather than a late gene and results in increased transcription/expression of *US11* (22, 23, 39). The product of *US11* is a component of the viral tegument and has multiple functions, all of which promote HSV-1 infection. *US11* expression leads to the inhibition of antiviral response mediators that include retinoic acid-inducible gene 1 (RIG-1), heat shock protein 90, nucleophosmin, OAS, and PKR, thus enhancing viral expansion (40–45). In summary, the enhanced expression of *US11*, together with the deletion of *UL34.5* and *US12*, allows T-VEC to replicate successfully in tumor cells by suppressing cellular antiviral response, while simultaneously promoting a robust immune response to infection, resulting in tumor-specific killing properties of this OV.

Although T-VEC is the first and only viral therapy currently approved by the FDA for treatment of unresectable melanoma lesions recurrent after initial surgery, there are limitations to its use and efficacy as a cancer treatment (46). These limitations derive from how it is administered and dosed, median time to response, effect on distant and visceral metastases, and the current requirement that it only be used as an adjuvant treatment.

When used as intended by the manufacturer, T-VEC must be injected directly into cutaneous, subcutaneous, and lymph node lesions multiple times for optimal activity (46). Moreover, administration of T-VEC is intended to be used on the largest lesions first, followed by the smaller lesions, until all lesions have been injected or the maximum injection volume of four milliliters is reached. As a result, patients may see evidence of regression of injected lesions, but disease progression may take place in untreated lesions (23). Additionally, realization of treatment benefits seems to be delayed with therapeutic responses occurring months after the prescribed dosing regimen is used. Because of this extended time-to-response period, many patients may see disease progression rather than regression (12). Thus, the combination of a maximum dosing volume and prolonged median time-to-response represent shortcomings of T-VEC therapy for melanoma.

In this light, the application of T-VEC for more generalized cancer therapy is under investigation by several groups (**Table 3**). As reviewed by Grigg et al, profound systemic regressions did not occur in T-VEC clinical trials involving visceral metastases, therefore, subjects with more advanced disease derived considerably less benefit from T-VEC therapy (23). Even so, 15% of measurable visceral metastases reduced in size by at least 50% in T-VEC-treated patients in a separate phase III clinical trial (12, 47).

**Table 3:** Current T-VEC Clinical Trials.

Cancer Type	NCT Number	Phase	Number of Subjects Enrolled	Intervention
**Breast Cancer**	NCT03802604	Phase I	28 (active)	Evaluate the efficacy of T-VEC with atezolizumab in subjects with breast cancer
	NCT03554044	Phase I	recruiting	Evaluate the efficacy of T-VEC with established chemotherapy or endocrine therapy in patients with Her2 negative breast cancer
	NCT02779855	Phase I and II	50 (active)	Evaluate the efficacy of T-VEC with paclitaxel in patients with triple negative breast cancer
**Colorectal Cancer**	NCT03256344	Phase I	36 (completed)	Intrahepatic injection of T-VEC with IV administered atezolizumab in triple negative breast cancer
	NCT03300544	Phase I	3 (active)	T-VEC in combination with 5-fluorouracil, leucovorin, oxaliplatin, capecitabine, and chemoradiation before surgery in treating patients with rectal cancer
**Liver Cancer**	NCT02509507	Phase I	127 (active)	T-VEC injected into liver tumors alone and in combination with systemic pembrolizumab
**Malignant Pleural Effusion**	NCT03597009	Phase I and II	1(terminated)	Administration of T-VEC into the intrapleural space of subjects with malignant pleural effusion through a pleurX catheter.
**Melanoma**	NCT03064763	Phase I	18 (active)	Administration of T-VEC by intralesional injection into patients with unresectable stage IIIB-IV malignant melanoma
	NCT03088176	Phase Ib	4 (active)	Administration of T-VEC by intralesional injection in conjunction with oral therapy with dabrafenib and trametinib
	NCT01740297	Phase Ib and II	217 (completed)	Administration of T-VEC in combination with ipilimumab
	NCT02366195	Phase II	112 (completed)	Dose response of intralesional injection of T-VEC into cutaneous, subcutaneous and nodal lesions.
	NCT04068181	Phase II	72 (active)	Administration of T-VEC in combination with pembrolizumab in patients with prior anti-PD-1 therapy for unresectable/metastatic melanoma
	NCT02211131	Phase II	150 (completed)	Administration of T-VEC followed by surgical resection of melanoma
	NCT02965716	Phase II	47	Combined T-VEC and pembrolizumab administration in patients with melanoma that progressed on anti-PD1/L1
	NCT02819843	Phase II	19 (active)	Administration of T-VEC with or without radiotherapy for cutaneous melanoma, Merkel cell carcinoma or other solid tumors.
	NCT03972046	Phase II	(withdrawn)	Administration of T-VEC in combination with FRAF/MEK inhibitor
	NCT02574260	Phase II	3 (completed)	Extension protocol for extended use of T-VEC in subjects participating in NCT00289016
	NCT00289016	Phase II	50 (completed)	Intratumoral injection of T-VEC in patients with stage IIIc and stage IV malignant melanoma
	NCT03842943	Phase II	28 (active)	Administration of pre-operative T-VEC injections combined with the neoadjuvant pembrolizumab
	NCT02263508	Phase III	713 (terminated)	Intratumoral injections of T-VEC and pembrolizumab
	NCT01368276	Phase III	31 (completed)	Treatment of tumors with GM-CSF and T-VEC-extension protocol of NCT00769704
	NCT00769704	Phase III	437 (completed)	Treatment of unresectable stage IIIb and IV melanoma with T-VEC compared to subcutaneous GM-CSF.
**Non-CNS Tumors**	NCT02756845	Phase I	15 (active)	Treatment of children 12-21yo with advanced non-CNS tumors with direct injection of tumors with T-VEC
**Non-Melanoma Skin Cancer**	NCT03458117	Phase I	26 (completed)	Intratumoral injection of T-VEC in patients with non-melanoma skin cancer
	NCT04163952	Phase I	5 (active)	IM-delivered T-VEC combined with panitumumab delivered IV to patients with advanced squamous cell carcinoma
	NCT03714828	Phase II	28 (recruiting)	Intralesional injection of T-VEC in patients with low-risk squamous cell carcinoma.
**Other**	NCT03747744	Phase I	18 (active)	Intratumoral injection of T-VEC followed by injection of CD1c^+^ myDC to subcutaneous, cutaneous, soft tissue metastases.
	NCT03555032	Phase I and II	15 (completed)	Administration of T-VEC by isolated limb perfusion (ILP) for treatment of melanoma and sarcoma
	NCT02014441	Phase II	61 (completed)	Intralesional injection of T-VEC in subjects with unresected, stage IIIB to IVM1c melanoma
**Pancreatic Cancer**	NCT03086642	Phase I	9 (active)	Endoscopically delivered T-VEC in patients with pancreas cancer refractory to at least one chemotherapeutic agent
	NCT00402025	Phase I	17 (competed)	Targeted delivery of T-VEC by endoscopic ultrasound in patients with irresectable pancreatic cancer
**Peritoneal Malignancies**	NCT03663712	Phase I	24 (recruiting)	Intraperitoneal T-VEC treatment in patients with peritoneal surface dissemination from gastrointestinal recurrent, platinum-resistance ovarian tumors
**Sarcoma**	NCT02453191	Phase I and II	30 (active)	T-VEC treatment combined with radiation therapy in patients with soft tissue sarcoma
	NCT04065152	Phase II	20 (recruiting)	T-VEC treatment of Kaposi sarcoma
	NCT03886311	Phase II	40 (recruiting)	Treatment of patients with advanced sarcoma with T-VEC, nivolumab and trabectedin
	NCT03069378	Phase II	60 (recruiting)	Combination therapy of T-VEC and pembrolizumab in patients with sarcoma
	NCT02923778	Phase II	40 (recruiting)	Combined T-VEC and radiation therapy in localized soft tissue sarcoma
	NCT03921073	Phase II	5 (active)	Intralesional injections of T-VEC in patients with advanced cutaneous angiosarcoma
**Squamous Cell Carcinoma**	NCT02626000	Phase I	36 (completed)	T-VEC in combination with pembrolizumab in patients with recurrent or metastatic squamous cell carcinoma of the head and neck

Lastly, findings by Kaufman et al. demonstrated that T-VEC therapy yielded significantly higher durable response rates across all disease stages compared to patients in the control group (47). This was particularly true for treatment-naïve patients who had not received any sort of prior treatment (12). This is significant because given that T-VEC is currently considered a novel immunotherapy, it is unlikely that patients will receive it as a first-line treatment. Therefore, in many patients under current treatment guidelines, diminished therapeutic outcomes using T-VEC are likely.

While there is evidence that T-VEC effectively promotes regression and necrosis of superficial melanoma lesions, it does not represent a complete therapy and has limited efficacy in patients who have visceral metastases. Hence, there is a need for continued research and development of other virus-based immunotherapies that are more broadly applicable. Many HSV-1 OV therapies are currently in clinical trials and may provide solutions to the limitations associated with T-VEC.

### HF10

HF10 is a naturally occurring mutant of HSV-1 undergoing clinical trials for use as an OV cancer treatment (48). Despite HF10’s ability to replicate efficiently in cells, its pathogenicity is highly attenuated in humans (49, 50). The genome of HF10 has insertions, deletions, and frameshift mutations affecting several genes. HF10 lacks functional expression of *UL43*, *UL49.5* and has only a single copy of both *UL56*, and *LAT* for which there are two in the wild-type virus. *UL52, UL53*, *UL54, UL55*, and *UL56*, are removed from their original positions, inverted, and reinserted later in the genome (**Figure 2**) (48). Because these mutations were not deliberate and methodical, it is difficult to ascertain their cumulative effect.

Individual consideration of each of the four genes that are functionally deleted provides clarification as to why HF10 may be superior to T-VEC for use as an OV therapeutic agent. For example, studies have shown that the bovine herpesvirus-1 (BHV-1) version of *UL49.5* encodes a protein that normally binds and degrades TAP in virus-infected cells leading to downregulation of MHC class I antigen presentation (51). Therefore, the loss of functional *UL49.5* in HF10 may similarly allow for maintenance of MHC I antigen presentation in infected human cells, resulting in a more robust adaptive immune response, similar to the effects derived from deletion of *US12* in T-VEC.

*LAT*, which encodes a long, noncoding RNA transcript, is functionally absent from the HF10 genome. Although *LAT* limits both establishment and reactivation from latency specifically in neurons, it appears to be less important for peripheral infections (17, 52). Mutational analysis of HSV-1 shows that viruses lacking *LAT* expression either fail to efficiently establish latency, or they cannot readily reactivate from latency, further increasing the safety of HF10 (53–55).

The absence of *UL56* results in reduction of the neuroinvasiveness and pathogenicity of HF10 compared to wild-type HSV-1 (56, 57). While *UL56* is not necessary for viral replication, viral strains lacking *UL56* are substantially less neuroinvasive and are unable to penetrate the central nervous system (CNS) (57, 58). *UL56* functionally increases the pathogenicity of HSV-1 by promoting the axonal transport of vesicles containing viral envelope glycoproteins through its interaction with KIF1A, a neuron specific kinesin. KIF1A plays an important role in the transport of synaptic vesicle precursors and in the axonal transport of pre-synaptic vesicles (59). The binding of *UL56* to KIF1A leads to neuronal cell dysfunction and, therefore, is partially responsible for the neuropathology of HSV-1 infection (56). Together, mutations in *LAT* and *UL56* in HF10 work to reduce viral neurovirulence and the likelihood of reactivation from latency, further enhancing the long-term safety of HF10 (60).

Unlike all other HSV-1 OVs that have entered clinical trials, *UL34.5* remains functionally intact in the HF10 genome (48). A potential drawback of the *IL34.5* loss-of-function mutants is that they replicate less efficiently, giving rise to lower viral yields as compared to wild-type virus, which could account for the limited efficacy demonstrated by T-VEC in clinical trials (12, 61, 62). It seems likely that functional ICP34.5 leads to high viral replication and the stronger antitumor effects of HF10 seen in clinical trials (63). Additionally, the duplications of *UL53*, *UL54*, and *UL55*, all of which are essential virus life cycle genes, could contribute to the high replication rate of HF10 (**Table 1**). Therefore, HF10 maintains tumor specificity and high-level replication.

High mitotic rates and the weakened interferon responses of tumor cells potentiate HF10’s candidacy as a cancer therapeutic. This is due to HF10’s superior ability to replicate and spread, as compared to wild-type HSV-1 strains (64, 65). Indeed, it has been shown to provoke a complete cytopathic effect and elicit a potent antitumor effect against a broad range of malignancies (66–68). Specifically, HF10 is able to produce a more vigorous bystander effect as compared to other HSV-1 variants (69). This is in alignment with studies that have shown the importance of intercellular trafficking and gap junctions for the production of this effect (69). Cancer cells typically have a dramatically reduced number of functional gap junctions allowing for more efficient tumor progression but increasing the difficulty for many OVs to produce the bystander phenomenon (70). Cancer cells decrease their gap junctions by altering their connexin expression, including Connexin 43 (Cx43). Cx43 is a protein that links adjacent cells’ cytoplasm. If the expression of Cx43 is suppressed, then gap junction activity is reduced or abrogated (71). However, HF10 appears to upregulate expression of Cx43 (72). This may allow HF10 to produce a more potent bystander effect through the formation of gap junctions.

HF10 has been shown to be safe in many dose-escalation phase I trials involving a variety of cancer types including recurrent and metastatic breast cancer, recurrent head and neck squamous cell carcinoma, unresectable pancreatic cancer, and melanoma (49, 50, 65, 72–74). A phase II trial has also been conducted to assess efficacy and safety of intratumoral injection of HF10 in combination with intravenous infusions of ipilimumab, a drug designed to boost T-cell responses. This trial revealed that HF10 in combination with ipilimumab is safe and well tolerated, with high antitumor efficacy (73). Additional phase II trials have been completed (NCT03153085, NCT02428036, NCT01017185, NCT02272855) or are currently underway (NCT03259425) for use of HF10 against melanoma. In total, the combined data from these clinical trials and preclinical studies illustrate multiple characteristics that make HF10 a superior OV therapy candidate when compared to other OVs that can be summarized in five key points: 1) high tumor selectivity, 2) high viral replication, 3) strong cytopathic effect, 4) potent bystander effect, and 5) vigorous antitumor effect against a variety of malignancies (66–69, 75–77). Therefore, HF10 shows great promise as a virus-based therapy and is likely to be broadly applicable, providing solutions to many limitations associated with T-VEC.

### HSV1716 (Seprehvir ^®^ or Seprehvec ^®^)

Having a relatively simple platform, HSV1716 was derived from a naturally occurring strain of HSV-1 containing only a spontaneous mutation resulting in the loss of functional neurovirulence-related *UL34.5*, making it similar to T-VEC (27, 78). In like fashion, the mutation renders HSV1716 incapable of replicating in the CNS and yet capable of replicating in and lysing dividing tumor cells (78).

The safety of this OV strain has been assessed in phase I and IIa trials for high-grade glioma (HGG), stage IV melanoma and mesothelioma (26, 79–81). HGG patients who experience recurrence of disease typically exhibit new lesions within approximately two centimeters of the original site of cancer growth. Harrow et al. injected HSV1716 into sites of healthy brain tissue directly adjacent to the region where HGG had been resected (26). In this way, lytic replication of HSV1716 could contain tumor spread. Although disease progression varied, no toxicity from the HSV1716 was observed in any of the study’s 12 subjects. Importantly, there was a significant increase in long-term survival post-resection in the OV treatment group. The potential of HSV1716 for increasing HGG survival is encouraging, but more trials need to be completed.

In a different application of HSV1716, Mackie et al. injected melanoma nodules with the OV, and performed immunohistochemical staining demonstrating that viral replication was restrained to tumor cells (80). Furthermore, in the three patients that received two or more doses of HSV1716, microscopic tumor necrosis was detectable. None of the patients exhibited any toxic effects due to the treatment, demonstrating an acceptable safety profile for HSV1716 and making it a potentially viable treatment for advanced melanoma.

In yet another phase I trial of HSV1716, Streby et al. investigated the safety of intratumoral injection of the OV in pediatric patients with non-CNS solid tumors (20). It was determined that single-dose intratumoral administration of HSV1716 is safe and well-tolerated in pediatric subjects with refractory non-CNS solid tumors. However, none of the subjects had a clinically measurable outcome. Therefore, the group suggested that this OV treatment should be used in a combination therapy or administered earlier in disease progression to allow it to develop an antitumor immune response (20). In fact, many pre-clinical trials have used HSV1716 in combination therapy showing synergistic effect (82–86). Uniquely, most of the subjects in this trial were HSV-1 seronegative, possibly because all of the subjects were children and had not been previously exposed to the virus. Since adults tend to be HSV-1 seropositive at a greater rate than this trial’s study population, the data could not be extrapolated to the general population.

A more recent study by Streby et al. explored the safety of HSV1716 applied intravenously. In this study they were unable to detect the OV in tumor biopsies, likely because the doses used were too low. Nevertheless, because it has the potential to reach all metastatic sites, the investigators remain optimistic about intravenous HSV1716 application at higher doses because no dose-limiting toxicities were observed (87). The absence of any observable anti-tumoral effect from the intravenous OV inoculation prompted the investigators to speculate that patients might receive greater benefit from combined intravenous and intratumoral administration, as such a dosing regimen could boost local immune responses within tumors.

In addition to these phase I trials, HSV1716 has also been used in a phase I/IIa trial investigating use of the OV for treatment of mesothelioma (MPM) patients (81). In this study, HSV1716 was well tolerated, with 50% of participants exhibiting disease stabilization and four out of twelve patients developing anti-tumor IgG. Intrapleural HSV1716 was well-tolerated and demonstrated an anti-tumor immune response in MPM patients. These results provide a rationale for further studies with this agent in MPM and in combination with other therapies

As one of the first OVs to be developed, HSV1716 has been used in multiple clinical trials, animal studies and *in vitro* studies that continue to generate invaluable data, which influence the design of other more sophisticated OVs such as T-VEC. Indeed, the safety profile of HSV1716 is excellent even if robust efficacy has not yet been demonstrated, thus providing a benchmark for future OV development either as standalone or part of combination cancer therapy. The repeated demonstration of HSV1716’s safety paves the way for further studies utilizing this OV to be performed, likely enabling the development of next-generation OVs.

### G207 & G47Δ

G207 is the first HSV-1 strain genetically engineered for treatment of intracerebral cancer to be used in clinical trials in North America (88). In contrast to T-VEC which was derived from wild-type HSV-1 strain JS1, this OV was derived from wild-type HSV-1 strain F (89). While G207 is modified differently than T-VEC, it does share the similarity that both copies of *UL34.5* are deleted, resulting in reduced neurotoxicity (89). Deletion of *UL34.5* in this HSV-1 variant also results in the alteration of LAT expression, leading to in the inability of the virus to establish latency (90).

G207 also has a *lacZ* insertion in *UL39*, leading to the inactivation of ICP6, a large subunit of the viral ribonucleotide reductase (**Figure 2**). ICP6 catalyzes conversion of ribonucleotides to deoxyribonucleotides, which is important for viral DNA replication. Therefore, it is required for efficient growth of the virus in nondividing cells, which do not produce a functionally equivalent enzyme like that produced in proliferating host cells (91–93). Consequently, functional deletion of ICP6 likely curtails G207 replication in quiescent cells, thereby preventing destruction of tissues adjacent to the tumor (91, 94). This makes the utility of this OV in cancer therapy clear (90). Other functions attributed to ICP6 include inhibition of apoptosis, establishment of latent infections, and reactivation from latency. Thus, loss of ICP6 as in this OV variant, contributes to both its safety and its efficacy in multiple ways (95, 96).

Markert et al. conducted multiple phase I trials to test the safety of G207 in the context of progressive, recurrent malignant glioma. Their original study was a dose escalation study in which the maximal tolerated dose was not achieved (88). In a follow-up study, they determined that intratumoral delivery of the OV before tumor resection followed by delivery into brain tissue surrounding the resection cavity, within one week of the first dose, was also safe (97). In a third trial, G207 OV therapy showed an excellent safety profile with none of the patients developing HSV encephalitis following intratumoral injections combined with radiation treatment (98). Additionally, these studies exhibited potential for clinical response in patients with progressive, recurrent, malignant glioma. Other phase I trials investigating the use of G207 in children are ongoing (NCT03911388, NCT02457845, NCT04482933). All three studies will investigate the use of G207 in children with recurrent or progressive brain tumors with or without radiation to enhance viral replication and an associated anti-tumor immune response.

In short, there is potential for G207 to play a vital role in combination therapy by intratumoral injection in glioblastoma, having demonstrated an acceptable safety profile in multiple trials. Compared to T-VEC and other OV therapies, its genetic composition differs due to the insertion of *lacZ* within *UL39*. The advantage of *lacZ* expression is that it potentially allows detection of viral replication in treated tumor tissues and any spread of the virus.

A third generation OV, G47Δ, was derived from G207 by deleting the gene encoding ICP47 (**Figure 2**), which prevents downregulation of MHC I, thus enhancing antitumor immune responses similar to T-VEC (**Table 2**) (99). This deletion also places *US11* under the control of the *US12* promoter, which may allow for higher replication capacity than OVs lacking *UL34.5*. Many studies utilizing animal or *in vitro* models have suggested great potential for G47Δ efficacy in killing tumor cells (100–106). To date, all clinical trials involving G47Δ have been conducted in Japan. In addition to demonstration of safety, this OV exhibited strong antitumor efficacy in patients with glioblastoma when used in a phase I-IIa trial (UMIN000002661) and a phase II trial (UMIN000015995) (107). Furthermore, clinical trials to investigate the safety of G47Δ in patients with recurrent or progressive olfactory neuroblastoma (UMIN000011636) and progressive malignant pleural mesothelioma (UMIN000034063) are underway. Significantly, a clinical trial using G47Δ to treat prostate cancer has also been completed (108).

The modifications to the G47Δ genome have given it a higher replication capacity than T-VEC and higher antitumor activity than its G207 parent virus. With improved therapeutic efficacy arising from increased replication and spread, this OV may represent the next stage in cancer therapy. Indeed, the architects of G47Δ, now called Teserpaturev, are seeking approval for Malignant Glioma therapy from Japan’s Ministry of Health (109).

### NV1020

NV1020, previously R7020, is a first-generation HSV-1 OV variant that is highly attenuated, having originally been developed as a herpes vaccine, albeit unsuccessfully (110). The construction of NV1020, described by Meignier, has a 15-kb deletion at the junction of the UL and US regions of the HSV-1 genome (**Figure 2**) where one of two copies of each of the genes encoding ICP0, ICP4, and ICP34.5 are located (111). In addition, this deletion removed one copy of *UL56.* ICP0 is a dispensable gene product, but at least one copy of the gene encoding ICP4, which blocks apoptosis and positively regulates several other HSV-1 genes, must be retained for viral replication. NV1020 also contains a 700-bp deletion that encompasses *TK* and the *UL24* promoter. HSV-1 UL24 has been shown to inhibit the activation of NF-ĸB, which together with interferon-response factor 3 (IRF3), triggers the host antivirus response *via* the cyclic GMP-AMP synthase viral DNA recognition pathway. Therefore, deletion of UL24 likely results in a greater innate immune response to this OV (**Table 2**) (112, 113). In place of the deletion at the junction of the UL and US regions, a 5.2-kb fragment of HSV-2 DNA and an exogenous *TK* have been inserted. Addition of the *TK* sequence guarantees that any potential NV1020 infection can be contained by TK-converted prodrugs such as acyclovir, adding a level of safety to this OV treatment.

In contrast to G207, NV1020 retains ICP6 and one copy of *UL34.5*, and for this reason was predicted to replicate more efficiently in tumor cells that have variable capacity to compensate for the loss of the ribonucleotide reductase (114). Further, investigators predicted that the maintenance of one copy of *UL34.5* in NV1020 likely allows for greater viral replication than G207. Indeed, it was determined in cell lines and *in vivo* animal experiments that NV1020, with improved therapeutic efficacy due to increased replication and spread, exerts greater cytotoxicity at lower multiplicities of infection (MOIs). The paradox of NV1020’s increased neurotoxicity potential set against an increased survival advantage at low MOIs of this OV led investigators to project that NV1020 might be advantageous for patients with more advanced cancer and larger tumors (114).

To date, NV1020 has been tested in at least two clinical trials, a phase I trial in colorectal cancer (NCT00012155) and a combined phase I/II trial (NCT00149396), both of which utilized hepatic artery infusion for liver-metastasized colorectal cancer patients who had failed their first line of chemotherapy (115, 116). Although adverse events were observed after administration in the phase I trial, most were mild to moderate and self-limiting, leading the investigators to conclude that NV1020 can be safely administered into the hepatic artery without significant effects on normal liver function (115). The second trial also integrated a dose escalation study, but it was followed by two cycles of chemotherapy for the cohort of subjects determined to have received the optimal dose. Once again, it was demonstrated that the treatment was minimally toxic, and a significant number of the study participants showed at least a partial response and/or stable disease. It was, therefore, concluded that the treatment may sensitize metastases to salvage chemotherapy, thus justifying performance of a phase II/III trial (116).

NV1020 retains one copy of *UL34.5*, suggesting a greater replication capacity than T-VEC. The fact that NV1020 was found to have greater replication and killing capacity than G207 in *in vitro* and animal studies justified pursuit of phase I and II trials (114). Although retention of one copy of UL34.5 likely allows for increased cytolytic activity over T-VEC, given the role of ICP34.5 in neurovirulence, the retention of this gene also suggests a potential risk of neurotoxicity when administered systemically or intracerebrally. However, the lack of NV1020-related adverse events in phase I and II clinical trials, including those measured by neurological examination suggests low risk of neurotoxicity and supports phase II/III trials (116).

### rQNestin34.5 & NG34

One of the downsides of the OV therapies utilizing HSV-1 variants that has been pointed out several times in this review is that they often have severely attenuated replication due to the loss of *ICP34.5* expression through deletion of both gene copies. Recognizing this issue, Kambara et al. engineered an OV that safely retains *UL34.5*. In rQNestin34.5, *UL34.5* has been reintroduced into the HSV-1 genome under the control of the glioblastoma multiforme (GBM) specific promoter, *nestin* (**Figure 2***)* (117). *Nestin*, normally active during embryogenesis, is shut down in the adult brain but is active in glioblastoma cells. Consequently, robust OV expression should occur only in GBM cells.

Researchers are currently recruiting for a phase I clinical trial using rQNestin34.5 in GBM patients (NCT03152318). Despite carrying a single copy of *UL34.5*, researchers hope that its re-engineered genome will confine expression of the gene to cancer cells and that neurotoxicity will be highly attenuated, thus mirroring the safety profile of *UL34.5*-lacking T-VEC.

In a resourceful workaround that could be applied more generally for the ICP34.5 neurotoxicity issue, the *nestin* promoter-controlled *UL34.5* was switched out with the human ortholog, *GADD34*. The new protein product, GADD34, expressed by this OV variant, known as NG34, mimics ICP34.5’s ability to dephosphorylate eIF2α by association with PP1, but it lacks a beclin 1 binding domain, effectively eliminating beclin 1-mediated neurotoxicity. To date, only animal studies have been performed with NG34, but greater tolerability compared to rQNestin34.5 has been demonstrated and could represent a viable evolutionary path of the parental OV if it performs well in clinical trials (118).

### M032

M032 is a second-generation oncolytic HSV variant that has been modified not only to take advantage of direct oncolytic activity but, also, to recruit inflammatory cells. This latter ability was imbued by incorporating *interleukin-12* (*IL-12*) into both sites where the gene encoding ICP34.5 was deleted to induce significant expression of this cytokine (**Figure 2** and **Table 2**). Transgene expression of IL-12 by an OV complements work performed with other vectors that yielded promising results in numerous other cancer therapy preclinical and clinical trials (119–124). Preclinical studies using an identical virus, except that it expresses murine IL-12, have shown this OV to be both safe and superior in efficacy to non-cytokine-expressing parental strains in various brain cancer models (125). This sets the stage for a phase I clinical trial (NCT02062827) that is currently recruiting for the treatment of recurrent/progressive glioblastoma.

With the insertion of a cytokine gene to enhance immune responses against OV-infected cells, M032’s design parallels that of T-VEC. The design similarities extend to deletion of the gene encoding ICP34.5 in both OVs, but the similarities end with the deletion of the gene encoding ICP47 in T-VEC. Therefore, it is difficult to project whether M032 will exhibit superior performance to T-VEC with respect to the treatment of malignancies. Nevertheless, expression of IL-12 could make this a more effective OV-based cancer treatment because of its ability to create an antitumor environment through enhancement of natural killer cell cytolytic activity. Additionally, IL-12 mediates production of IFN-γ, polarizing T helper cells towards a cell-mediated response. Moreover, it has been demonstrated that IL-12 is antiangiogenic, thereby inhibiting tumor growth (119, 122, 126).

## Discussion

There is an ongoing need for development of efficacious cancer therapies that will increase survival of patients with resistant malignancies, and OV treatment shows considerable promise. As a component of combination therapy regimens utilizing standard-of-care (SOC) treatments, there is mounting preclinical and clinical evidence that OV treatments can boost overall therapeutic efficacy against a variety of malignancies (73, 82–84, 127, 128). Incorporation of OVs into treatment regimens introduces the potential for significant dose reductions without compromising tumor cell-killing capacity. In turn, toxicity associated with chemo- and radiotherapy can be minimized. In this review of the many OV therapies currently in development, only HSV-1 variants in clinical trials or approved for clinical use were discussed.

OV treatments display potential for extending life expectancy in patients with various refractory cancers, however, completion of subsequent phases of these trials must be performed in order to determine efficacy and establish patient safety. Furthermore, many aspects of OV therapy require further research to optimize mode of adminstration to establish efficacy against different types of malignancies and to hone the specificity and safety properties of each engineered OV variant.

Use of OVs for cancer treatment ideally results in two separate but related therapeutic effects. The first is that infection of tumor cells by an OV leads to direct death of the cancer cells as a part of their lytic lifecycle. The viral progeny released upon lytic destruction can subsequently infect other cancer cells to perpetuate killing of more cells. However, solid tumors are known to extensively manufacture extracellular matrix (ECM) (129). Therefore, ECM-mediated inhibition of virus spread throughout tumors is an area of active exploration within the OV therapy field (130, 131). Several groups have incorporated genes for ECM-remodeling proteins into various OVs that have shown promise in preclinical models (132–136). Improved antitumor efficacy has been observed when OVs expressing chondroitinase, hyaluronidase, relaxin, and decorin were used (132–137). Since studies using OVs containing a single transgene that regulates connective tissue remodeling have shown some success, it is possible that delivery of a combination of these genes by OVs will be required for maximum tissue penetration and tumor regression.

Release of cancer antigens from lysed cells leads to the second therapeutic effect- the induction of an adaptive immune response that kills residual local and distant tumor cells. To this end, establishment of highly robust immune responses against cancer cells has been the focus of many OV engineering efforts. Earliest efforts using HSV-1 as an OV were directed at modifying the virus so that its capacity to evade host immune responses would be attenuated. The deletion of *UL34.5* from many HSV-1 OV strains is an example. This deletion reduces neurovirulence and allows enhanced immune responses due to the fact that wild-type virus expression of ICP34.5 inhibits DC maturation and antigen presentation (26, 27, 32–34). Other gene deletions in HSV-1 OVs that result in more complete immune responses against virus-infected cells include removal of *US12* and *UL49.5*, both of which negatively affect the function of TAP (51).

Excellent safety profiles of early HSV-1 OVs have been clinically established, so recent efforts are aimed at increasing efficacy by addition of transgenes that enhance physiological responses directed towards OV-infected cells. For example, HSV-1 has been engineered to include transgenes that allow the virus to synergize with SOC treatments such as chemotherapy and radiation. To address inadequate chemotherapeutic responses Braidwood et al, developed an HSV-1 OV that contains the enzyme, nitroreductase, which converts the prodrug, CB1954, into an active alkylating chemotherapeutic agent. Use of this OV together with CB1954 enhanced tumor cell killing *in vitro* and improved survival in preclinical cancer models (138).

Another SOC treatment commonly used for treating cancer is radiation therapy. Observations have been made wherein radiation positively affects viral replication and, therefore, has the potential to contribute to OV therapeutics. Conversely, radiation therapy can be enhanced with OV treatment. This approach was taken by Quigg, et. Al. wherein they inserted a transgene coding for the noradrenaline transporter into an HSV-1 OV (139). This modification enabled targeted radiotherapy of HSV-infected cells expressing the membrane symporter because they accumulated the iodine-131-labeled noradrenaline analog, metaiodobenzylguanidine (MIBG). The combination of the OV and radiolabeled MIBG led to decreased tumor growth and increased survival in an animal model relative to either agent given alone (140).

Both approaches result in concentration of conventional therapeutic agents in and around infected cells with the goal of killing tumor cells with high precision while minimizing toxicity to healthy cells. However, replacement of toxic therapeutic agents by Ovs that have been engineered to promote physiological responses robust enough to destroy the tumor is the ultimate goal. One way this might be accomplished is to boost immune responses against tumor cells by modifying Ovs to deliver therapeutic payloads. Currently, insertion of cytokine genes that promote inflammation and subsequent bystander killing of tumor cells into Ovs is a common approach. The incorporation of *GM-CSF* into T-VEC, and *IL-12* into M032 are notable examples. Numerous other cytokine transgenes have been incorporated into Ovs as well as inhibitory receptors and bispecific T cell engagers with varying degrees of success (141–145). Given the large HSV-1 genome containing multiple accessory genes and the seemingly endless array of possible transgenes that could be incorporated, this approach represents a significant source of as yet untapped potential.

T-VEC’s reduced replication and restricted mode of delivery by intra-tumoral application has led investigators to take a step back and consider the possible use of unattenuated viruses. Replication-competent viruses might be safely used if they can be engineered to infect only specific cancer cells, sparing non-cancerous surrounding cells. Therefore, reliance on specificity rather than attenuation for OV therapy may be a superior strategy.

By and large, retargeting is accomplished by modification of the viral receptor, gD. Cancer cells employ a variety of strategies to enhance their own survival. For example, many cancer cells express the IL-13 receptor to promote an anti-inflammatory environment. Zhou et al. created an HSV-1 OV that expresses a chimeric gD receptor that contains IL-13 sequences to target cells expressing the IL-13 receptor (146). In an alternative approach, multiple groups have replaced portions of the gD gene sequence with that of single-chain immunoglobulin receptors. These were directed against tumor associated antigens such as human epidermal growth factor receptor (EGFR), epithelial cell adhesion molecule (EpCAM) and human epithelial growth factor receptor 2 (HER2), thereby retargeting Ovs to recognize a variety of tumor cell types (147–150). In each of these retargeted HSV-1 Ovs, gD was modified but *UL34.5* was retained, preserving full replication capacity while providing greater specificity. This strategy parallels the previously mentioned rQNestin34.5, which has *UL34.5* under a *nestin* promoter to specifically kill glioblastoma cells.

Avoiding immune responses is another area of OV development being actively researched. Although OVs can be modified to specifically target tissues, administration of unaccompanied virus has one significant downside in which neutralizing antibodies that either already exist in circulation or arise as a result of treatment can drastically reduce viral titers. Viral clearance by neutralizing antibodies has hampered the use of T-VEC and other OVs in clinical trials for treatment of visceral metastases by systemic or intravenous application (12, 23, 73). Consequently, a number of ‘ghosting’ techniques have been developed that allow delivery of the virus in a manner that prevents it from being exposed to the body’s immune defenses.

Ghosting OVs can involve ‘Trojan horse’ or ‘hitchhiking’ cell-based delivery methods. Systemic administration of OVs by Trojan horse is one of the most commonly explored techniques, likely because it mimics naturally occurring microbial strategies to avoid the immune system, such as that used by Flaviviruses. It is well established that tumor cells tend to traffic *in vivo* to sites of origin or tumor cell distribution, making them attractive potential delivery vehicles (151). To avoid seeding *de novo* tumor growth by these carriers, strategies have employed: lethal irradiation of tumor cells prior to delivery, use of allogeneic or xenogeneic cells, and/or use of inducible suicide programs (152, 153). Similarly, mesenchymal stem cells (MSCs) can serve as cell-based OV delivery vehicles since they readily migrate to sites of inflammation, injury, ischemia, and home to tumor microenvironments (154–158). Additional advantages gained from use of MSCs include ease of isolation from patients and growth in culture, reduced immunogenicity, and high metabolic activity that allows for increased viral replication and subsequent viral yields at tumor sites (155, 158). These cell-based delivery mechanisms avoid immune depletion of virus as demonstrated by higher viral titers and improved tumor killing in animal models.

Hitchhiking commonly involves the use of immune cells, which naturally home to tumors as a part of host defense. HSV-1 OVs adsorbed onto the surface of tumor-specific lymphocytes followed by systemic injection resulted in greater cytotoxicity of tumor cells and increased survival in a disseminated tumor mouse model, supporting the use of hitchhiking as a second viable mechanism for OV delivery (159).

As an alternative to cell-based carriers, the viral envelope can be modified to mask it from host immune defenses. This has been done in multiple ways, including addition of inhibitory regulators of phagocytosis or biodegradable polymers to OV envelopes (160, 161). Both of these techniques enable OVs to evade host defenses, thus allowing the virus to persist longer so as to reach more tumor cells.

Since only a small portion of OV carriers typically reach their intended destination, researchers have gone a step further by combining directed targeting with Trojan horse strategies. Using a mouse model, Muthana et al. employed magnetic resonance imaging (MRI) to steer systemically injected macrophages, loaded with magnetic beads and a payload of OV (HSV1716), from the blood to specific tumor sites. This combination strategy resulted in increased tumor macrophage infiltration and a reduction in tumor burden and metastases (162). This work supports the possibility for real-time image-guided trafficking of carrier cells containing targeted OVs to ensure viral delivery at tumor sites and improve clinical outcomes.

OV therapies show great promise towards the goal of defeating cancer with minimal collateral pathology. In particular, patients who are refractory to current SOC treatments may derive enormous benefit from OV therapy. Of the many viruses being studied for OV development, HSV-1 is particularly suitable for the task. The HSV-1 OV, T-VEC, is the only FDA-approved OV therapy in the United States and has already been shown to be safe and effective in the treatment of melanoma. However, there is room for improvement of this or any of the other HSV-1 OV therapies currently being developed. New research has made significant progress in creating OV technologies that can be applied to the HSV-1 platform to provide greater cancer specificity and augment tumor killing with minimal toxicity.

## Author contributions

HS: Originated the idea for the review, wrote the paper outline, the Introduction and other several sections, prepared the final format for **Figure 1**; ZC: Wrote several sections, prepared **Table 3**; B: Wrote several sections, and performed reference research well as reference list assembly; TS: Wrote several sections, created **Table 2** and oversaw editing; DB: Wrote several sections, created **Table 1** and oversaw editing. All authors contributed to the article and approved the submitted version.

## Conflict of interest

The authors declare that the research was conducted in the absence of any commercial or financial relationships that could be construed as a potential conflict of interest.

## Publisher’s note

All claims expressed in this article are solely those of the authors and do not necessarily represent those of their affiliated organizations, or those of the publisher, the editors and the reviewers. Any product that may be evaluated in this article, or claim that may be made by its manufacturer, is not guaranteed or endorsed by the publisher.
